# The Framework for Human Host Immune Responses to Four Types of Parasitic Infections and Relevant Key JAK/STAT Signaling

**DOI:** 10.3390/ijms222413310

**Published:** 2021-12-10

**Authors:** Tsung-Han Wen, Kuo-Wang Tsai, Yan-Jun Wu, Min-Tser Liao, Kuo-Cheng Lu, Wan-Chung Hu

**Affiliations:** 1 Department of Anatomical Pathology, Taipei Tzu Chi Hospital, Buddhist Tzu Chi Medical Foundation, New Taipei City 231, Taiwan; ashin1258@gmail.com; 2 Department of Medical Research, Taipei Tzu Chi Hospital, Buddhist Tzu Chi Medical Foundation, New Taipei City 231, Taiwan; kwtsai6733@gmail.com; 3 Department of Pediatrics, Taoyuan Armed Forces General Hospital, Taoyuan City 325, Taiwan; wuyanjun@aftygh.gov.tw (Y.-J.W.); liao-ped804h@yahoo.com.tw (M.-T.L.); 4 Division of Nephrology, Department of Medicine, Fu-Jen Catholic University Hospital, School of Medicine, Fu-Jen Catholic University, New Taipei City 242, Taiwan; kuochenglu@gmail.com; 5 Department of Clinical Pathology & Medical Research, Taipei Tzu Chi Hospital, Buddhist Tzu Chi Medical Foundation No. 289, Jianguo Road, Xindian District, New Taipei City 231, Taiwan

**Keywords:** immune response, parasitic infection, eosinophils, mast cells, basophils, protozoa, helminths, parasitic insects, JAK/STAT

## Abstract

The human host immune responses to parasitic infections are complex. They can be categorized into four immunological pathways mounted against four types of parasitic infections. For intracellular protozoa, the eradicable host immunological pathway is TH1 immunity involving macrophages (M1), interferon gamma (IFNγ) CD4 T cells, innate lymphoid cells 1 (NKp44+ ILC1), CD8 T cells (Effector-Memory4, EM4), invariant natural killer T cells 1 (iNKT1) cells, and immunoglobulin G3 (IgG3) B cells. For intracellular protozoa, the tolerable host immunological pathway is TH1-like immunity involving macrophages (M2), interferon gamma (IFNγ)/TGFβ CD4 T cells, innate lymphoid cells 1 (NKp44- ILC1), CD8 T cells (EM3), invariant natural killer T 1 (iNKT1) cells, and immunoglobulin A1 (IgA1) B cells. For free-living extracellular protozoa, the eradicable host immunological pathway is TH22 immunity involving neutrophils (N1), interleukin-22 CD4 T cells, innate lymphoid cells 3 (NCR+ ILC3), iNKT17 cells, and IgG2 B cells. For free-living extracellular protozoa, the tolerable host immunological pathway is TH17 immunity involving neutrophils (N2), interleukin-17 CD4 T cells, innate lymphoid cells 3 (NCR- ILC3), iNKT17 cells, and IgA2 B cells. For endoparasites (helminths), the eradicable host immunological pathway is TH2a immunity with inflammatory eosinophils (iEOS), interleukin-5/interleukin-4 CD4 T cells, interleukin-25 induced inflammatory innate lymphoid cells 2 (iILC2), tryptase-positive mast cells (MCt), iNKT2 cells, and IgG4 B cells. For ectoparasites (parasitic insects and arachnids), the eradicable host immunological pathway is TH2b immunity with inflammatory basophils, chymase- and tryptase-positive mast cells (MCct), interleukin-3/interleukin-4 CD4 T cells, interleukin-33 induced nature innate lymphoid cells 2 (nILC2), iNKT2 cells, and immunoglobulin E (IgE) B cells. The tolerable host immunity against ectoparasites and endoparasites is TH9 immunity with regulatory eosinophils, regulatory basophils, interleukin-9 mast cells (MMC9), thymic stromal lymphopoietin induced innate lymphoid cells 2, interleukin-9 CD4 T cells, iNKT2 cells, and IgA2 B cells. In addition, specific transcription factors important for specific immune responses were listed. This JAK/STAT signaling is key to controlling or inducing different immunological pathways. In sum, Tfh is related to STAT5β, and BCL6 expression. Treg is related to STAT5α, STAT5β, and FOXP3. TH1 immunity is related to STAT1α, STAT4, and T-bet. TH2a immunity is related to STAT6, STAT1α, GATA1, and GATA3. TH2b immunity is related to STAT6, STAT3, GATA2, and GATA3. TH22 immunity is associated with both STAT3α and AHR. THαβ immunity is related to STAT1α, STAT1β, STAT2, STAT3β, and ISGF. TH1-like immunity is related to STAT1α, STAT4, STAT5α, and STAT5β. TH9 immunity is related to STAT6, STAT5α, STAT5β, and PU.1. TH17 immunity is related to STAT3α, STAT5α, STAT5β, and RORG. TH3 immunity is related to STAT1α, STAT1β, STAT2, STAT3β, STAT5α, STAT5β, and ISGF. This categorization provides a complete framework of immunological pathways against four types of parasitic infections. This framework as well as relevant JAK/STAT signaling can provide useful knowledge to control allergic hypersensitivities and parasitic infections via development of vaccines or drugs in the near future.

## 1. Introduction

Host immune responses to parasitic infections are complex. Parasites include protozoa, helminths, and insects. Previously, the author proposed a framework for all the known host immunological pathways and their roles in the immune responses against four specific types of pathogens and the corresponding four specific types of hypersensitivities [1,2]. In this framework, the author included TH1, TH2, TH3, TH9, TH17, TH22, Tfh, Treg, and Tr1 to describe their roles in eradicable immunity or tolerable immunity in detail [3,4,5,6,7]. Tfh cells are the T helper cells that initiate eradicable immunity with B cell antibody class switch to IgG. Treg cells are the T helper cells that initiate tolerable immunity with B cell antibody class switch to IgA. TH1 immune response is the eradicable host immunity against intracellular pathogens including intracellular bacteria, fungi, or protozoa (intracellular microorganisms). TH1-like immune response is the tolerable host immunity against intracellular pathogens including intracellular bacteria, fungi, or protozoa (intracellular microorganisms). Both TH1 and TH1-like immunities are related to type 4 delayed-type hypersensitivity. TH2 immune response is the eradicable host immunity against helminths and insects. TH9 immune response is the tolerable host immunity against helminths and insects. Both TH2 and TH9 immunities are related to type 1 allergy hypersensitivity. TH22 immune response is the eradicable host immunity against extracellular bacteria, fungi, and protozoa (extracellular microorganisms). TH17 immune response is the tolerable host immunity against extracellular bacteria, fungi, and protozoa (extracellular microorganisms). Both TH22 and TH17 immunities are related to type 3 immune complex hypersensitivity. THαβ (Tr1) immune response is the eradicable host immunity against viruses. TH3 immune response is the tolerable host immunity against viruses. Both THαβ and TH3 immunities are related to type 2 antibody-dependent cytotoxic hypersensitivity. However, the TH2 host immunological pathway against parasites can be further divided into TH2a and TH2b to react to different pathogens, such as helminths and insects, respectively. Here, we extend the framework and propose a new framework of host immunological pathways for four types of parasitic infection. Host immunological pathways against parasites are determined primarily by the location of the infection. After identifying the location of the parasitic infection, the host immune system can attack these parasites with different effector cells using different strategies.

STAT proteins are master regulators of host immunological pathways [1]. After binding to receptors, different cytokines activate different JAK/STAT signaling pathways to trigger different immunological pathways in response to different pathogens [3,4,5,6,7,8]. STAT proteins are transcription factors, and they can compete with each other, leading to different host immunological pathways against specific pathogens. JAK/STAT signaling is the major downstream signaling of multiple cytokine receptors. Cytokine receptors can activate different JAK/STAT signaling pathways to mediate different immune functions. Here, we summarize these JAK/STAT signaling pathways and their roles in immune function as well as diseases, especially parasitic infections.

Eradicable immunological pathways are initiated by follicular helper T cells (Tfh) [9]. These T cells can secrete interleukin 21 to induce a B cell antibody class switch from IgM to IgG [10]. IgG is characteristic of eradicable host immune reactions. IL-21 receptor activation can induce JAK1/JAK3 signaling to activate STAT5β [11]. The magnitude of JAK1 activation by interleukin-21 is higher than that by interleukin-2 or interleukin-15 [12]. STAT1, STAT3, and STAT5β are the main transcription factors activated by interleukin 21 to mediate the function of follicular helper T cells. Afterwards, BCL6 is upregulated by STAT5β to initiate the function of follicular helper T cells. The eradicable immunological pathways can be categorized into four groups (TH1, TH22, TH2, and THαβ) based on the subtypes of IgG antibodies and different pathogens.

The regulatory immunological pathway is initiated by regulatory CD4 T cells (Tregs) [9]. When the pathogen infection is diffuse involving an organ, strong eradicable immunity with IgG response will cause fulminant organ damage. Thus, milder regulatory immunity with IgA response is initiated to mildly control the pathogens to co-exist with these pathogens. For example, the mucosal immunity in the gastroenteric tract is the regulatory immunity to co-exist with *Escherichia coli* and other intestinal pathogens. If the host cannot eradicate the infection, Tregs will be triggered only to limit the infection and immune reaction. FOXP3 is a key transcription factor of regulatory CD4 T cells. STAT5 transcription factors, especially STAT5α, can upregulate FOXP3. TGFβ is the main cytokine that mediates Treg cell function. This can cause B cell antibody class switching to IgA antibodies. Interleukin-2 also plays a critical role in the development of regulatory T cells. Interleukin-2 receptors can activate JAK1 and JAK3 to phosphorylate STAT5α and STAT5β and mediate Treg function [13,14]. Compared to interleukin-21, JAK3 signaling induced by interleukin-2 is stronger. Based on the type of pathogen, regulatory immunological pathways can also be categorized into four groups (TH1-like, TH17, TH9, and TH3).

## 2. Host Immunological Pathways for Different Types of Parasitic Infections

### 2.1. Intracellular Protozoa and TH1/TH1-like Immunity

**Figure 1:**
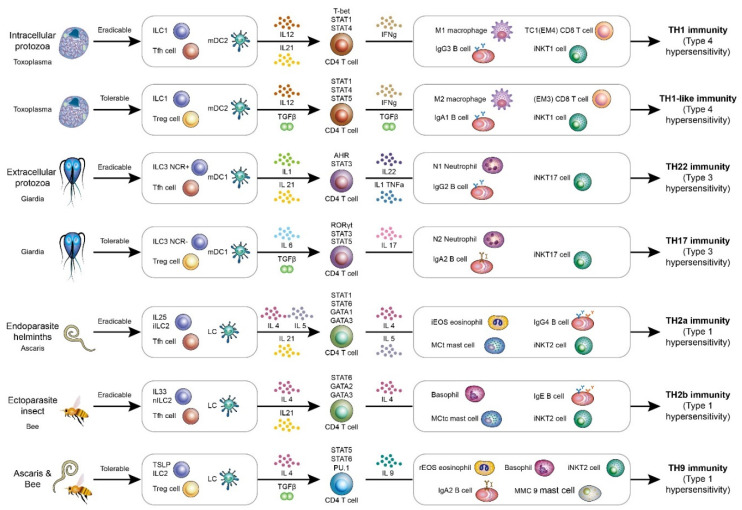
A framework of host immunological pathways against parasitic infections. The host immunological pathways can be divided into Tfh-mediated eradicable (IgG related) or Treg-mediated tolerable (IgA related) immune responses. For intracellular micro-organisms including intercellular protozoa, the eradicable immunity is TH1 immunity, and the tolerable immunity is TH1-like immunity. For extracellular micro-organisms including extracellular protozoa, the eradicable immunity is TH22 immunity, and the tolerable immunity is TH17 immunity. For endoparasites including helminths, the eradicable immunity is TH2a immunity. For ectoparasites including insects, the eradicable immunity is TH2b immunity. For endoparasites and ectoparasites, the tolerable immunity is TH9 immunity.

For intracellular protozoa, the host immunological pathway is a TH1 immune response involving macrophages (M1), interferon gamma (IFNγ) CD4 T cells, CD8 T cells (CD28+CD27− EM4, or Tc1), invariant natural killer T1 (iNKT1 cells), and IgG3 B cells [15,16,17]. Innate lymphoid cells 1 (NKp44+CD103− ILC1) is the immune cells helping to initiate TH1 immune reaction [18]. The type 1 ILCs, which are positive for NKp44 (natural cytotoxic receptor), are the typical cytotoxic type 1 innate lymphoid cells secreting IFNγ in response to interleukin-12 or interleukin-18. These type 1 NKp44+ ILCs are located in intra-epithelial locations [18,19]. Type 2 myeloid dendritic cells (CD141+ mDC2) are the main antigen-presenting cells that initiate TH1 immune reaction. The driven cytokine for TH1 immunity is interleukin-12. IFNγ-secreting CD4 T helper cells are the central mediators in the TH1 immunity. Macrophages with IFNγ stimulation will be activated to become inflammatory M1 macrophages. IFNγ can activate induced nitric oxide synthetase (iNOS) to kill the intercellular organisms in the phagolysosomes of macrophages [20]. The key transcription factors for TH1 immunity are STAT1, STAT4, and T-bet. Interleukin-12 can activate STAT4, and interferon gamma can activate STAT1. IFNg can activate cytotoxic CD8 T cells. CD8 can be categorized into four subgroups according to the expression of CD27 and CD28. CD28+ CD8 T cells are for eradicable immunity, and CD28− CD8 T cells are for tolerable immunity. The CD27 molecule is used for anti-viral immunity, but not for anti-intracellular bacteria, fungi, or protozoa [21]. Cytotoxic T cells 1 (TC1), or so-called Effector-Memory CD8 T cells 4 (CD28+CD27- EM4), are used for TH1 anti-intracellular bacteria, fungi, or protozoa immunity. Cytotoxic T cells 2(Tc2), or so-called Effector-Memory CD8 T cells 1 (CD28+CD27+ EM1), are used for THαβ anti-virus immunity [22]. IFNγ can induce a B cell class switch to human IgG3 or corresponding murine IgG2b antibody [23]. Invariant natural killer T cells are immune cells recognizing lipid or glycolipid antigens from pathogens. iNKT1 cells are the subtypes of iNKT cells against intracellular bacteria, fungi, or protozoa. TH1 immunity is the host immune response to intracellular pathogens. The intracellular location is more important than the pathogen type. Thus, TH1 immunity can be triggered to defend against intracellular bacteria, fungi, and protozoa. Activated macrophages are the key effector cells that digest intracellular bacteria, fungi, and protozoa. Intracellular protozoa are categorized into the parasite groups. Intracellular protozoa, including *Plasmodium*, *Leishmania*, *Toxoplasma*, *Babesia*, and *Cryptosporidium*, can all trigger a TH1 host immune response [24,25,26,27,28,29,30,31,32,33,34]. Intracellular bacteria, such as *Chlamydia*, and intracellular fungi, such as *Histoplasma*, can also trigger TH1 immunity. This is the intracellular protozoa-eradicable host immune response. Type 4 hypersensitivity (delayed-type hypersensitivity) is related to TH1 immunity. The whole host immunological pathways against parasitic infections are summarized in Table 1 and Figure 1.

**Figure 2:**
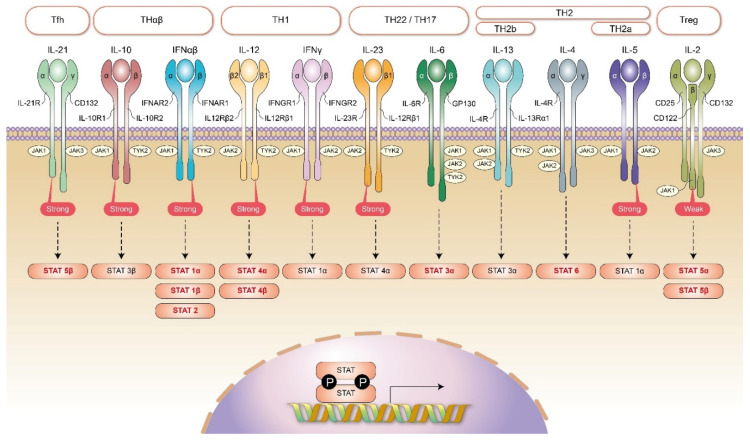
JAK/STAT signaling and the framework of host immunities. In Tfh immune reaction, the key mediating JAK molecules are JAK1 (stronger) and JAK3. They activate downstream STAT5β. In Treg immune reaction, the key mediating JAK molecules are JAK1 and JAK3 (stronger). They activate downstream STAT5α and STAT5β. In TH1 immunity, the key cytokine receptors are interleukin-12 and IFNγ. The key mediating JAK molecules for interleukin-12 receptor are JAK2 and TYK2 (stronger). They activate downstream STAT4α and STAT4β. The key mediating JAK molecules for IFNγ receptor are JAK1 (stronger) and JAK2. They activate downstream STAT1α. In TH2 immunity (including TH2a and TH2b immunities), the key cytokine receptor is interleukin-4 receptor. The key mediating JAK molecules for interleukin-4 receptor are JAK1, JAK2, and JAK3. They activate downstream STAT6. In addition, in TH2a immunity, an additional interleukin-5 receptor is needed. The key mediating JAK molecules for interleukin-5 receptor are JAK1 and JAK2 (stronger). They activate downstream STAT1α. Additionally, in TH2b immunity, an additional interleukin-13 receptor is needed. The key mediating JAK molecules for interleukin-13 receptors are JAK1, JAK2, and TYK2. They activate downstream STAT3α. In TH22/TH17 immunity, the key cytokine receptors are interleukin-6 and interleukin-23. The key mediating JAK molecules for interleukin-6 receptor are JAK1, JAK2, and TYK2. They activate downstream STAT3α. The key mediating JAK molecules for interleukin-23 receptor are JAK2 (stronger) and TYK2. They activate downstream STAT4α. In THαβ immunity, the key cytokine receptors are IFNα/β and interleukin-10. The key JAK molecules for IFNα/β are JAK1 and TYK2 (stronger). They activate downstream STAT1α, STAT1β, and STAT2. The key JAK molecules for interleukin-10 receptor are JAK1 (stronger) and TYK2. They activate downstream STAT3β.

The TH1 immunological pathway includes macrophages (M1), CD8 T cells, iNKT1 cells, IFNγ-producing CD4 T cells, and IgG3 B cells. The TH1 immune reaction is a host response against intracellular pathogens, including intracellular bacteria, protozoa, and fungi. TH1 immunity is driven by interleukin-12. Type 1 innate lymphoid cells (ILC1) and type 2 myeloid dendritic cells (mDC2) also play important roles in initiating the TH1 immunological reaction. The signaling for transducing the TH1 immune response by interleukin-12 is mediated by JAK2/TYK2 [35]. TYK2 signaling induced by interleukin-12 is stronger than that induced by interleukin-23. IFNγ activates STAT1α via JAK1/JAK2 [36]. This signaling further activates downstream STAT1α and STAT4 transcription factors, which also activates T-bet transcription. These transcription factors contribute to the TH1 immunological pathway. The TH1 immunological pathway is associated with type 4 delayed-type hypersensitivities, such as tuberculosis reaction, type 1 diabetes, and multiple sclerosis. The JAK/STAT signaling in each host immunological pathway is summarized in Figure 2.

For immune tolerance to intracellular protozoa, the host mounts a TH1-like immune response. The effector cells for TH1-like immunity are macrophages (M2), IFNγ/TGFβ CD4 T cells, Effector-Memory CD8 T cells 3 (CD28−CD27− EM3), iNKT1 cells, and IgA1 B cells [16,17]. Innate lymphoid cell 1 (NKp44− ILC1) is an immune cell that helps to initiate TH1-like immune response. The regulatory type 1 non-cytotoxic innate lymphoid cells without a natural killer receptor marker (NKp44) are located in mucosa to promote TH1-like immunity [37,38]. Regulatory CD4 T helper cells (Treg) play a critical role in initiating tolerable immune responses. Treg cells can activate STAT5A and STAT5B transcription factors via interleukin-2 [39]. Both STAT5A and STAT5B play non-redundant roles in the regulatory or tolerable immunological pathways. Regulatory dendritic cells (DCreg) also help antigen presentation in the tolerable TH1-like immunity. The effector cells for TH1-like immunity are the alternate activated macrophages (M2 macrophages), which is transformed by TGFβ and IFNγ. Alternative activated macrophages M2 are the principal cells mediating the TH1-like immunity to intracellular pathogens. IFNγ/TGFβ CD4 T cells are the central CD4 T helper cells in mediating TH1-like immunity. Effector-Memory CD8 T cells 3 (CD28−CD27−EM3) are the main cytotoxic T cells targeting the intracellular pathogens in this tolerable immune response [15]. It is worth noting that Effector-Memory CD8 T cells 2 (CD28-CD27+ EM2) are the main cytotoxic T cells against viruses in tolerable host immune response. TGFβ produced from regulatory T helper cells can cause B cell antibody class switch into IgA. Additionally, IgA has two subtypes: IgA1 is in serum and IgA2 is in the mucosal surface. For intracellular bacteria, fungi, or protozoa, IgA1 is the major type of IgA antibody in this tolerable host immunity. The key transcription factors for TH1-like immunity are STAT1, STAT4, and STAT5. STAT4 and STAT1 can be activated by interleukin-12 and IFNγ, respectively. Like TH1 immunity, iNKT1 is a natural killer T cell against intracellular pathogen lipid antigens. TH1-like immunity is a chronic immune tolerance to intracellular pathogens, including intracellular bacteria, protozoa, and fungi. Chronic infections with intracellular protozoa usually trigger the TH1-like immunological pathway. Type 4 hypersensitivity (delayed-type hypersensitivity) is related to TH1-like immunity.

The TH1-like immunological pathway includes alternatively activated macrophages (M2), CD8 T cells, iNKT1 cells, IFNγ and TGFβ-producing CD4 T cells, and IgA1-producing B cells. The TH1-like immune reaction is a host response against intracellular pathogens, including intracellular bacteria, protozoa, and fungi. TH1-like immunity is driven by interleukin-12 and TGF-β. Type 1 innate lymphoid cells (ILC1) and type 2 myeloid dendritic cells (mDC2) also play important roles in initiating the TH1 immunological reaction. This signaling activates downstream STAT5α/β, STAT1α, and STAT4 transcription factors. These transcription factors contribute to the TH1 immunological pathway. TH1-like immunological pathway is associated with type 4 delayed-type hypersensitivities, such as tuberculosis reaction, type 1 diabetes, and multiple sclerosis.

### 2.2. Extracellular Protozoa and TH22/TH17 Immunity

For free-living extracellular protozoa, the eradicable host immunological pathway is TH22 immunity with neutrophils (N1), interleukin-22 CD4 T cells, iNKT17 cells, and IgG2 B cells. Innate lymphoid cells 3 (NCR+ ILC3) help to initiate TH22 immunity. Natural cytotoxic receptor (NCR) is positive for the type 3 innate lymphoid cells in initiating the TH22 immunological pathway by secreting interleukin-22. Type 1 myeloid dendritic cells (mDC1) are the antigen-presenting cells for initiating TH22 host immunity. Pro-inflammatory cytokines, including interleukin-1, TNF-α, or interleukin-6, can initiate the TH22 immunity against extracellular organisms. These pro-inflammatory cytokines can activate STAT3 transcription factor. Inflammatory type 1 neutrophils (N1) are the major effector cells of the TH22 host immunological pathway. Interleukin-22-producing CD4 T helper cells play central roles in mediating TH22 immunity. The key transcription factors for TH22 immunity are STAT3 and Aryl Hydrocarbon Receptor (AHR). The interleukin-22/AHR/STAT3 axis of CD4 T helper cells plays a key role in mediating TH22 immunity. iNKT17 cells are the major NKT cells against lipid or glycolipid antigens from extracellular organisms. IgG2 is the human B cell-producing antibody, which can form an immune complex with soluble extracellular organisms. The TH22 cytokines interleukin-1α and interleukin-1β are shown to induce B cell isotype switch into human IgG2 antibody [40]. TH22 immunity is the host immune response to extracellular protozoa, bacteria, and fungi. It is worth noting that extracellular location determines the host immunological pathway, which is more important than whether the pathogen is bacteria, fungi, or protozoa. Neutrophils can use neutrophil extracellular traps and kill these extracellular free-living pathogens. These extracellular free-living protozoa include *Trypanosoma*, *Amoebae*, *Giardia*, and *Trichomonas* [41,42,43,44,45,46,47,48]. These pathogens can induce TH22 host immunity. Extracellular bacteria, such as *Escherichia coli*, and extracellular fungi, such as *Aspergillus*, can also trigger TH22 host immune reactions. Type 3 hypersensitivity (immune complex mediated) is related to TH22 immunity.

The TH22 immunological pathway includes neutrophils (N1), interleukin-22-producing CD4 T cells, iNKT17 cells, and IgG2 B cells. The TH22 immune reaction is a host response against extracellular bacteria, protozoa, and fungi. TH22 immunity is driven by TNF-α or interleukin-1. Type 3 innate lymphoid cells (ILC3) and type 1 myeloid dendritic cells (mDC1) also play important roles in initiating the TH22 immunological reaction. The signaling for transducing the TH22 immune response is mediated by JAK1/JAK2/TYK2, which are downstream signaling molecules of interleukin-6 [49,50,51]. The signaling activates downstream STAT3 transcription factors. In addition, the aryl hydrocarbon receptor (AHR) is a key mediator of TH22 immunity. Moreover, interleukin-23 can activate STAT4α via JAK2/TYK2 signaling [52]. This also helps trigger the TH22/TH17 immune reaction. TH22 immunological pathway is associated with type 3 immune complex hypersensitivities such as Arthus reaction and rheumatoid arthritis.

The immune tolerance pathway against extracellular protozoa, fungi, and bacteria is TH17 immunity. The effector cells of TH17 immunity include neutrophils (N2), interleukin-17 CD4 T cells, iNKT17 cells, and IgA2 B cells. Innate lymphoid cells 3 (NCR− ILC3) without the expression of natural cytotoxic receptor (NCR−) are the innate immune cells that help to initiate TH17 immunity by secreting interleukin-17. Regulatory CD4 T helper cells (Treg) play a critical role in initiating tolerable immune responses. Treg cells can activate STAT5A and STAT5B transcription factors via interleukin-2. Both STAT5A and STAT5B play non-redundant roles in the regulatory or tolerable immunological pathways. TGFβ plus interleukin-6 can trigger the TH17 immunological pathway. Regulatory dendritic cells (DCreg) also help antigen presentation in the tolerable TH17 immunity. Regulatory type 2 neutrophils are the effector cells for TH17 immunity. Interleukin-17-producing CD4 T helper cells play central roles in mediating TH17 immunity. The interleukin-17/RORγt/STAT3 axis is the central axis in TH17 immune reaction. The subtype of invariant NKT cells against extracellular organisms’ lipid or glycolipid antigens is the iNKT17 cell. TGFβ produced from regulatory T helper cells can cause B cell antibody class switch into IgA. The subtype IgA2 is in the mucosal surface. For extracellular bacteria, fungi, or protozoa, IgA2 is the major type of IgA antibody in this tolerable host immunity. The key transcription factors for TH17 immunity are STAT3, STAT5, and RORγt. The TH17 immune reaction is a chronic immune tolerance to extracellular free-living protozoa. Type 3 hypersensitivity (Immune complex mediated) is related to TH17 immunity.

The TH17 immunological pathway includes neutrophils (N2), interleukin-17-producing CD4 T cells, iNKT17 cells, and IgA2 B cells. The TH17 immune reaction is a host response against extracellular bacteria, protozoa, and fungi. TH17 immunity is driven by TNF-α or IL-6 plus TGF-β. Type 3 innate lymphoid cells (ILC3) and type 1 myeloid dendritic cells (mDC1) also play important roles in initiating the TH17 immunological reaction. The signaling for transducing the TH17 immune response is mediated by JAK1/JAK3 and JAK1/JAK2/TYK2. The signaling activates downstream STAT3α and STAT5α/β transcription factors. In addition, RORγt is also a key mediator of TH17 immunity. The TH17 immunological pathway is associated with type 3 immune complex hypersensitivities, such as Arthus reaction and rheumatoid arthritis.

### 2.3. Helminths (Endoparasites) and Eradicable TH2a Immunity

For helminths (endoparasites), the eradicable host immunological pathway is TH2a immunity with inflammatory eosinophils, interleukin-5/interleukin-4 CD4 T cells, tryptase-positive mast cells (MCt), iNKT2 cells, and IgG4 B cells. The meaning of endoparasites is parasites located in our bodies. Inflammatory innate lymphoid cells 2 (Interleukin-25 induced iILC2) help to initiate TH2a immune response [53,54]. Interleukin-25 can help innate lymphoid cells 2 to produce more interleukin-4, interleukin-5, and interleukin-13, and, in particular, stimulate eosinophil activation. While interleukin-4, interleukin-5, and interleukin-13 can all be up-regulated by interleukin-25 stimulation, interleukin-5 and interleukin-13 are mostly stimulated [55,56]. Additionally, interleukin-5 is strongly associated with eosinophil activation. The inflammatory innate lymphoid cells can help the host to defend itself against helminth infection initially. Langerhans cells are antigen-presenting cells that help to present antigens to initiate TH2 immune reaction [57]. Inflammatory eosinophils (iEOS) are the major effector cells that use IgG4-mediated antibody-dependent cellular toxicity to attack the helminth tegument [58]. IgG4 antibody is usually associated with eosinophilia. IgG4 subtype is the smallest amount of all IgG antibodies and accounts for 3% of all antibodies in the blood. Tryptase-positive mast cells are the mast cell subtypes in TH2a immunity. MCt secretes interleukin-5 and responds to platelet activating factor (PAF), a potent eosinophil chemoattractant and activator [59]. Interleukin-4/interleukin-5-producing CD4 T cells play central roles in mediating TH2a immunity, and interleukin-4 can activate STAT6 to initiate TH2 immunity. Type 2 invariant natural killer T cells (iNKT2) are the iNKT cell subtypes against helminth lipid or glycolipid antigens. This TH2a pathway belongs to TH2 immunity and is a subtype. The letter “a” in TH2a means “acid”, which is derived from the name of eosinophils. STAT6, STAT1, GATA1, and GATA3 are key transcription factors in TH2a immunity, which up-regulate interleukin-5 and the function of eosinophils [60,61,62,63]. Helminths (endoparasites) that can induce TH2a immunity with eosinophilia include *Ascaris*, hookworms, tapeworms, pinworms, filarial worms, *Toxocara*, and *Strongyloides* [64,65,66,67,68,69,70,71,72,73]. However, several helminths can also induce IgE antibodies, so this immune response is a subtype of the TH2 immune response. Type 1 hypersensitivity (allergy) is related to TH2a immunity, and it is a IgG4 dominant type 1 hypersensitivity.

The TH2 immunological pathway includes eosinophils (iEOS), basophils, mast cells, interleukin-4/interleukin-5-producing CD4 T cells, iNKT2 cells, and IgG4/IgE B cells. The TH2 immune reaction is a host response against extracellular helminths and insects. TH2 immunity is driven by interleukin-4. Type 2 innate lymphoid cells (ILC2) and Langerhans cells play important roles in initiating the TH2 immunological reaction. The signaling for transducing the TH2 immune response is mediated by JAK2/JAK3 [74,75]. This signaling activates the downstream STAT6 transcription factor. In addition, GATA transcription factors are vital to the TH2 immunological pathway. The TH2 immune response can be further divided into two subtypes: TH2a and TH2b. TH2a immunity acts against helminths with eosinophils (iEOS), mast cells, interleukin-5-producing CD4 T cells, iNKT2 cells, and IgG4 B cells. Interleukin-5 activates STAT1α via JAK1/JAK2 [76]. Compared to interferon gamma, JAK2 signaling induced by interleukin-5 is stronger.

### 2.4. Parasitic Insects and Arachnids (Ectoparasites) and Eradicable TH2b Immunity

For insects (ectoparasites), the eradicable host immunological pathway is TH2b immunity with inflammatory basophils, chymase- and tryptase-positive mast cells (MCct), interleukin-3/interleukin-4 CD4 T cells, iNKT2 cells, and IgE B cells. Ectoparasites means that these insects are located in our bodies’ outer skin surface. Nature innate lymphoid cells 2 (interleukin-33 induced nILC2) help to initiate TH2b immune reaction [56,77]. Langerhans cells are antigen-presenting cells responsible for antigen presentation in TH2b immunity [57]. The major effector cells of TH2b immunity are basophils and chymase- and tryptase-positive mast cells (MCct). MCct secretes interleukin-4 and interleukin-13 and has a C5a receptor to respond to C5a, an anaphylatoxin that induces IgE-mediated anaphylaxis. Interleukin-33 can also be produced by mast cells to mediate IgE-dominant allergic reaction. Circulating basophils and resident mast cells have the same characteristics. IgE antibody is the least represented antibody in humans, and it only accounts for 0.05% in peripheral blood. This can be explained by the fact that insect or arachnid infection is a rare event compared to other bacteria or virus infections. The letter “b” in TH2b means “base”, which is derived from the name of basophils. STAT6, GATA2, and GATA3 are key transcription factors for TH2b immunity, which up-regulate the function of basophils [60,61]. Interleukin-3/interleukin-4-producing CD4 T helper cells play central roles in mediating TH2b immunity. Interleukin-4 can activate the TH2 transcription factor STAT6. The subtypes of invariant natural killer T cells are iNKT2 cells combating insect or arachnid lipid or glycolipid antigens. IgE can cause the physical expelling of insects (ectoparasites) via skin itchiness, skin wheal with toxin dilution, rhinorrhea, mucus formation and secretion, nausea/vomiting, bronchoconstriction, and increased bowel movement. Basophil accumulation is usually noted at the site of insect bites or dwelling. This can explain why people are allergic to shrimps or crabs with the up-regulation of IgE antibodies. However, these IgE-mediated mechanisms can also expel helminths in the lungs or intestines. Thus, this immune response (TH2b) is a subtype of the TH2 immune response. The bites of parasitic arachnids and insects, including those of ticks, fleas, and mosquitos, can induce a TH2b immune reaction [78,79,80,81,82]. The stings of non-parasitic insects, such as bees and wasps, also induce a TH2b immune reaction. Type 1 hypersensitivity (allergy) is related to TH2b immunity, and it is IgE-dominant type 1 hypersensitivity.

TH2b immunity acts against insects with basophils, mast cells, interleukin-4/interleukin-13-producing CD4 T cells, iNKT2 cells, and IgE B cells. Both TH2a and TH2b immune responses are related to the activation of STAT6. However, GATA transcription factors differ between the two subtypes of immune reactions. In TH2a immunity, GATA1 and GATA3 play crucial roles, whereas in TH2b immunity, GATA2 and GATA3 play crucial roles. STAT3 also plays an important role in TH2b immunity. Interleukin-13 can activate STAT3α via JAK1, JAK2, and TYK2 [83]. The TH2 immunological pathway is associated with type 1 allergic hypersensitivities, such as asthma, allergic rhinitis, and atopic dermatitis [84].

### 2.5. Parasites and Tolerable TH9 Immunity

The TH9 host immunological pathway is a chronic immune tolerance response to parasites (endoparasites and ectoparasites). The main effector cells of the TH9 immunological pathway include regulatory eosinophils, regulatory basophils, mast cells (MMC9), interleukin-9 CD4 T cells, iNKT2 cells, and IgA2 B cells [58,85]. Thymic stromal lymphopoietin (TSLP)-induced innate lymphoid cells 2 help to initiate TH9 immunity [56,86]. Type 2 innate lymphoid cells driven by TSLP produce interleukin-9 and interleukin-13. Thus, type 2 innate lymphoid cells can be categorized into three subtypes: IL-25 driven, IL-33 driven, and TSLP driven. The three subtypes are responsible for different host immunological pathways against parasites including TH2a, TH2b, and TH9. Regulatory CD4 T helper cells (Treg) play a critical role in initiating tolerable immune responses. Treg cells can activate STAT5A and STAT5B transcription factors via interleukin-2. Both STAT5A and STAT5B play non-redundant roles in regulatory or tolerable immunological pathways. TGFβ plus interleukin-4 can trigger the TH9 immunological pathway. Regulatory dendritic cells (DCreg) also contribute to the antigen presentation in tolerable TH17 immunity. Regulatory eosinophils and regulatory basophils are the effector cells for TH9 immunity. Interleukin-9-producing mast cells (MMC9) are the mast cell subtype responsible for TH9 immunity. Interleukin-9-producing CD4 T helper cells play central roles in mediating TH9 immunity. The subtype of invariant NKT cells against extracellular organisms’ lipid or glycolipid antigens is iNKT2 cell. TGFβ produced from regulatory T helper cells can cause B cell antibody class switch into IgA. The subtype IgA2 is in the mucosal surface. For extracellular parasites, IgA2 is the major type of IgA antibody in this tolerable host immunity. STAT6, STAT5, and PU.1 are key transcription factors for TH9 immunity to up-regulate interleukin-9 [87]. The TH9 driven cytokine intereleukin-4 can activate STAT6 to mediate parasite immunity. Type 1 hypersensitivity (allergy) is related to TH9 immunity, and it is a chronic tolerable immune reaction. Chronic asthma is an example of this TH9 immunological pathway.

The TH9 immunological pathway includes regulatory eosinophils (rEOS), basophils, mast cells (MMC9), interleukin-9-producing CD4 T cells, iNKT2 cells, and IgA2 B cells. The TH2 immune reaction is a host response against extracellular helminths and insects. TH9 immunity is driven by IL-4 and TGF-β (from Tregs). Type 2 innate lymphoid cells (ILC2) and Langerhans cells play important roles in initiating the TH9 immunological reaction. The signaling for transducing the TH2 immune response is mediated by JAK2/JAK3 and JAK1/JAK3. This signaling activates downstream STAT6 and STAT5α/β transcription factors. In addition, the PU.1 transcription factor is also vital for the TH9 immunological pathway. The TH9 immunological pathway is associated with type 1 allergic hypersensitivities, such as asthma, allergic rhinitis, and atopic dermatitis.

## 3. Other Host Immunological Pathways: Immunity against Viruses

The THαβ immunological pathway includes NK cells (NK1), interleukin-10-producing CD4 T cells, iNKT10 cells, and IgG1 B cells [88,89]. The THαβ immune reaction is an eradicable host response against viruses. THαβ immunity is driven by IFNα/β or IL-10. Innate lymphoid cells (ILC10) and plasmacytoid dendritic cells play important roles in initiating THαβ immunological reactions. The signaling for transducing the THαβ immune response is mediated by JAK1/TYK2 [90,91]. This signaling activates downstream STAT1α, STAT1β, and STAT3β transcription factors. In addition, IRF is also a key mediator of THαβ immunity [92]. JAK1 signaling induced by interleukin-10 is stronger than that induced by interferon α/β. The THαβ immunological pathway is associated with type 2 antibody-dependent cellular cytotoxic hypersensitivities, such as systemic lupus erythematous.

The TH3 immunological pathway includes NK cells (NK2), interleukin-10/TGFβ-producing CD4 T cells, iNKT10 cells, and IgA1 B cells. The TH3 immune reaction is a tolerable host response against viruses. TH3 immunity is driven by IFNα/β or interleukin-10 plus TGF-β. Innate lymphoid cells (ILC10) and plasmacytoid dendritic cells play important roles in initiating the TH3 immunological reaction. The signaling for transducing the TH3 immune response is mediated by JAK1/JAK3 and JAK1/TYK2. The signaling activates downstream STAT1α, STAT1β, STAT2, STAT3β, and STAT5α/β transcription factors. In addition, IRF is a key mediator of TH3 immunity. The TH3 immunological pathway is associated with type 2 antibody-dependent cellular cytotoxic hypersensitivities, such as systemic lupus erythematous.

## 4. Future Perspectives

This host immunological framework against parasitic disorders as well as relevant key JAK/STAT signaling can provide insights to develop drugs or vaccines to control parasitic infections and allergic hypersensitivities. TH1/TH1-like is the host immune reaction against intracellular micro-organisms. IFNγ is the key cytokine of TH1/TH1-like immunity to suppress intracellular protozoa. Thus, we could use IFNγ to treat malaria or toxoplasma infections. Previous studies also showed that IFNγ can totally kill malarial pathogens, especially during early infection [93]. Literature also showed that IFNγ can eliminate toxoplasma [94]. Thus, IFNγ can be used as a therapeutic agent to treat intracellular protozoa including malaria or *Toxoplasma*. Vaccines against intracellular protozoa should be able to induce IgG3 in TH1 immune response successfully. TH22/TH17 is the host immune response against extracellular micro-organisms. Interleukin-22 and interleukin-17 are the key cytokines in TH22 or TH17 immune responses. Literature showed that interleukin-22 can suppress extracellular protozoa including *Trichomonas* [44]. Literature also showed that interleukin-17 can control extracellular protozoa such as giardiasis [45]. Thus, interleukin-22/interleukin-17 can be used as therapeutic agents to treat extracellular protozoa including trichomonas or giardiasis. Vaccines against extracellular protozoa should be able to induce IgG2 in TH22 immune response. TH2/TH9 is the host immune response against parasites (endoparasites and ectoparasites). Interleukin-4 and interleukin-9 are the key cytokines in TH2/TH9 immunity to inhibit parasites including helminths and insects. Interleukin-4 can suppress intestinal nematode parasites including *Strongyles* or *Trichinella* [95]. Interleukin-9 can also enhance host resistance to intestinal nematodes such as *Trichuris* infection [96]. So, interleukin-4 and interleukin-9 can be used as therapeutic agents to treat infections of helminths or insects. Vaccines against helminths should be able to induce IgG4 in TH2 immunological pathway. In addition, TH2 immunity can be divided into TH2a immunity and TH2b immunity. Because immune reaction against helminths infection belongs to TH2a immunity with IgG4 antibody production. Thus, it is important to initiate TH2a immune reaction against helminths infections. Interleukin-5 is the key cytokine in TH2a immunity which can activate eosinophils. Thus, we can use interleukin-5 as a therapeutic agent to treat helminths infections. TH2b immunity with IgE antibody response is mainly against infections cause by insects. Thus, when we develop a vaccine against helminths, it is better to induce a TH2a immunity with IgG4 antibody response than to induce a TH2b immunity with IgE antibody response. It is worth noting that we should consider to use eradicable or tolerable cytokines used to treat above infections. When the parasitic infection is too diffuse and extensive such as diffuse infection of toxoplasma, we should use cytokines with tolerable immunity to mildly control the infection to avoid overt organ damage. Thus, our framework against parasitic diseases is very informative for drug or vaccine development for these infections. Besides, the relevant JAK/STAT signaling is also very useful for drug development strategies. JAK inhibitors such as abrocitinib, upadacinib, baricitinib, and gusacitinib have been developed to control allergic diseases including atopic dermatitis [97]. Other JAK inhibitors including ruxolitinib and deglocitinib are also under development to treat atopic dermatitis or food allergy which is associated with parasitic immune responses. It is important to identify allergic disease is TH2a or TH2b immune reaction. For example, allergic asthma is mainly induced by antigens from dust mites, parasitic insects, so TH2b is more likely to be the immune-pathogenesis of asthma. Basophils, IgE, and interleukin-13 play major roles in the TH2b immunopathogenesis. Previous studies using anti-interleukin-5 monoclonal antibody to treat allergic asthma. Even they found out that eosinophil counts decreased after anti-interleukin-5 antibody treatment, the asthmatic symptoms of these patients were not alleviated or only mild-moderate reduction in eosinophilic asthma [98,99]. In non-eosinophilic asthma, the effect of anti-interleukin-5 antibody is doubtful. This could be because that eosinophils, IgG4, and interleukin-5 are more important to TH2a immunity. Thus, therapeutic agents targeting TH2a immune reaction in asthma may not induce favorable effects. Previous studies also found out that anti-interleukin-13 antibody is more important to alleviate symptoms of allergic asthma [100]. Another review article also pointed out IgE antibody is more important than eosinophil in the immune-pathogenesis of asthma [101]. Thus, our immunological framework against parasitic infections which distinguish TH2a and TH2b immune response can be vital for controlling allergic diseases including asthma. Our framework is very informative to provide hints to control parasite infections as well as allergic hypersensitivities.

## 5. Conclusions

This framework describes the immunological pathways of the human host response to four types of parasitic infections. Intracellular protozoa induce TH1/TH1-like immunity; extracellular protozoa induce TH22/TH17 immunity; endoparasites (helminths) induce TH2a eradicable immunity; and ectoparasites (parasitic insects and arachnids) induce TH2b eradicable immunity. TH9 immunity is a tolerable immune response to endoparasites and ectoparasites. In addition, different JAK/STAT signaling is the key in each host immunological pathway against parasitic infections. When we determine the different frameworks for the host immunological pathways against intracellular protozoa, extracellular protozoa, endoparasites (helminths), and ectoparasites (insects), we should use different vaccine or drug development strategies to control these infections. Malarial pathogens are intercellular protozoa. Thus, we can use IFNγ from TH1/TH1-like immunity to control malarial infection. The host immune responses against parasites are related to hypersensitivities, including allergic reaction, such as asthma or atopic dermatitis. Once we determine the host immunities against parasitic infections, we can develop better vaccines or therapeutic agents to control these parasites, as well as these hypersensitivities. Drugs targeting master JAK/STAT signaling can also be developed to treat parasitic infections or hypersensitivities, including allergic responses, which are either IgE-dominant (controlling STAT3α and STAT6) or IgG4-dominant (controlling STAT1α and STAT6) reactions.

## Figures and Tables

**Table 1:** Summary of host immunological pathways against parasites.

Immune Pathways	Cytokines	Transcription Factors	Innate Lymphoid Cells	Effector Cells	CD4 T Cells	B Cells	NKT Cells	Pathogens	Autoimmunity
TH1 eradicable immunity	IFNg, IL-12	STAT1, STAT4, T-bet	ILC1	Macrophages M1	IFNg CD4 T cells	IgG3	iNKT1	Intracellular protozoa, bacteria, and fungi	Type 4 DTH
TH1-like tolerable immunity	IFNg, TGFβ	STAT1, STAT4, STAT5	ILC1	Macrophages M2	IFNg/TGFβCD4 T cells	IgA1	iNKT1	Intracellular protozoa, bacteria, and fungi	Type 4 DTH
TH22 eradicable immunity	IL-1, IL-6, TNFα	STAT3, AHR	ILC3 NCR+	Neutrophils N1	IL-22 CD4 T cells	IgG2	iNKT17	Extracellular protozoa, bacteria, and fungi	Type 3 immune complex
TH17 tolerable immunity	IL-6, TGFβ	STAT3, STAT5, RORγt	ILC3 NCR-	Neutrophils N2	IL-17 CD4 T cells	IgA2	iNKT17	Extracellular protozoa, bacteria, and fungi	Type 3 immune complex
TH2a eradicable immunity	IL-4, IL-5	STAT6, STAT1, GATA1/3	IL-25 iILC2	Eosinophils (iEOS), mast cells (MCt)	IL-5 CD4 T cells	IgG4	iNKT2	Helminths	Type 1 allergy IgG4 related
TH2b eradicable immunity	IL-4, IL-13	STAT6, STAT3, GATA2/3	IL-33 nILC2	Basophils, mast cells (MCct)	IL-4 /IL-13 CD4 T cells	IgE	iNKT2	Insects	Type 1 allergy IgE related
TH9 tolerable immunity	IL-4, TGFβ	STAT6, STAT5, PU.1	TSLP ILC2	Eosinophils (rEOS), basophils, mast cells (MMC9)	IL-9 CD4 T cells	IgA2	iNKT2	Helminths and Insects	Type 1 allergy

## Data Availability

Not applicable.
